# Heat Stress Pre-Exposure May Differentially Modulate Plant Defense to Powdery Mildew in a Resistant and Susceptible Barley Genotype

**DOI:** 10.3390/genes12050776

**Published:** 2021-05-19

**Authors:** Ildikó Schwarczinger, Judit Kolozsváriné Nagy, Lóránt Király, Klára Mészáros, Judit Bányai, Viola Kunos, József Fodor, András Künstler

**Affiliations:** 1 Centre for Agricultural Research, Plant Protection Institute, ELKH, 15 Herman Ottó Str., H-1022 Budapest, Hungary; schwarczinger.ildiko@atk.hu (I.S.); nagy.judit@atk.hu (J.K.N.); fodor.jozsef@atk.hu (J.F.); kunstler.andras@atk.hu (A.K.); 2 Centre for Agricultural Research, Agricultural Institute, ELKH, 2 Brunszvik Str., H-2462 Martonvásár, Hungary; meszaros.klara@atk.hu (K.M.); banyai.judit@atk.hu (J.B.); kunos.viola@atk.hu (V.K.)

**Keywords:** barley, BAX inhibitor, combined stress resistance, heat stress, pathogenesis-related genes, plant defense, powdery mildew infection, reactive oxygen species, respiratory burst oxidase homologue

## Abstract

Heat stress negatively affects barley production and under elevated temperatures defense responses to powdery mildew (*Blumeria graminis* f. sp. *hordei,* Bgh) are altered. Previous research has analyzed the effects of short-term (30 s to 2 h) heat stress, however, few data are available on the influence of long-term exposure to heat on powdery mildew infections. We simultaneously assessed the effects of short and long term heat pre-exposure on resistance/susceptibility of barley to Bgh, evaluating powdery mildew infection by analyzing symptoms and Bgh biomass with RT-qPCR in barley plants pre-exposed to high temperatures (28 and 35 °C from 30 s to 5 days). Plant defense gene expression after heat stress pre-exposure and inoculation was also monitored. Our results show that prolonged heat stress (24, 48 and 120 h) further enhanced Bgh susceptibility in a susceptible barley line (MvHV118-17), while a resistant line (MvHV07-17) retained its pathogen resistance. Furthermore, prolonged heat stress significantly repressed the expression of several defense-related genes (*BAX inhibitor-1*, *Pathogenesis related-1b* and *Respiratory burst oxidase homologue F2*) in both resistant and susceptible barley lines. Remarkably, heat-suppressed defense gene expression returned to normal levels only in MvHV07-17, a possible reason why this barley line retains Bgh resistance even at high temperatures.

## 1. Introduction

Plants are sessile organisms; therefore, a fast and efficient response to abiotic or biotic stresses is a key for their survival. When attacked by, e.g., pathogens, plants employ a variety of preformed and inducible defenses to prevent or at least limit infections. The first line of inducible plant defenses consists primarily of the pathogen-associated molecular pattern (PAMP) recognition system conferring a basal resistance (PAMP-triggered immunity, PTI) to a wide range of pathogens [1,2]. If the PAMP system fails to recognize the pathogen as an invader, susceptibility may develop, unless a second line of plant defense is induced by pathogen-secreted effectors when they are recognized by plant resistance (R) proteins (effector-triggered immunity, ETI) [1,3]. ETI is often associated with a localized programmed cell/tissue death (PCD) at infection sites called the hypersensitive response (HR) [4]. The signaling processes leading to an HR involve, e.g., the accumulation of reactive oxygen species (ROS). ROS like superoxide (O_2_^−^ or hydrogen peroxide, H_2_O_2_) have a dual role during plant defense, as high ROS concentrations confer inhibition of invading pathogens along with PCD of infected plant cells (HR), while low ROS concentrations act as signals inducing antioxidants and pathogenesis-related (PR) genes and proteins in plant tissues adjacent to infection sites [5,6,7,8,9]. PR genes/proteins are induced by both pathogen infections and abiotic stresses but an early, enhanced induction occurs during incompatible host–pathogen interactions (i.e., resistance), as compared to compatible interactions [10], suggesting a role during disease resistance. Although several PR proteins exhibit antimicrobial activities (e.g., degradation of fungal cell walls via glucanase and chitinase activities), a direct functional role in defense could not be demonstrated for all PR proteins [10,11]. The PR1-b protein may contribute to resistance to fungal pathogens possibly by binding sterols in fungal membranes [12,13].

Powdery mildew fungi are obligate biotrophic pathogens that grow and reproduce only in living cells of infected plants by obtaining nutrients through specialized feeding structures called haustoria [14]. These ascomycete fungi comprise more than 700 species that regularly cause serious economic losses mostly in dicot crops [15]. In the 1990s, the majority of fungicides were applied in order to control powdery mildew diseases [16]. Barley powdery mildew, *Blumeria graminis* f. sp. *hordei* (D.C.), Golovin ex Speer f. sp. *hordei* Em. Marchal (Bgh), is one of the very few of these pathogens that can infect a monocot grass species, barley (*Hordeum vulgare* L.) [17,18]. Barley is the fourth cereal crop in relevance worldwide [19] and environmental stresses, e.g., heat stress, have a significant impact on its production [20]. Analysis of climate records has shown an increasing risk of exposure to heat stress that could result in the reduction of cereal yields up to 0.5 t/ha by 2050 [21].

High temperatures interfere with various interconnected signaling pathways in plant cells, causing, e.g., an initially enhanced ROS production that contributes both to antioxidant induction (controlling ROS) and elevated expression of heat shock protein (HSP) genes encoding molecular chaperones that stabilize/protect, e.g., antioxidant enzymes. In addition, heat-induced extracellular Ca^2+^ influxes across plasma membranes activate heat shock transcription factors (HSFs) that regulate HSP expression, and Ca^2+^ is also responsible for activation of ROS-producing, disease resistance-associated NADPH oxidases. Importantly, the proper functioning of HSPs and antioxidant enzymes has been associated with the development of heat tolerance in several plant species (see [22,23] and references within).

While high temperature has negative or positive effects on plant pathogens as well [23,24], temperature elevation is expected to favor both the emergence of new pathogens and the occurrence and severity of epidemics [25]. Plants in nature must face many different stresses simultaneously and biotic and abiotic stressors are acting in concert, both having a significant impact on plant health [23,26]. In addition, the interplay of abiotic and biotic factors may influence plant pathogen interactions and the outcome of a given infection [27,28]. In fact, most identified plant defense responses are altered under elevated temperatures, regardless of the plant and pathogen species [23]. Overall, a growing number of studies have shown that heat stress can affect the plant defense response in different ways, however, it can be concluded that heat stress has usually negative effects on key plant resistance mechanisms [23]: for example, higher mean temperatures observed over an experimental period of six years in wheat, correlated with elevated susceptibility to the fungus *Cochliobolus sativus* [29]. In cereals, the effects of temperature and other environmental conditions on powdery mildew infections have been reviewed by [30]. The optimum temperature for development of powdery mildew is 20 °C [31] but the range at which it can infect and grow is wide. The upper limit for infection is about 30 °C [32]. In spring barley, the primary leaves are highly susceptible to Bgh, but when grown at 30 °C, the pathogen fails to infect its host [33].

The duration of heat stress is also an important factor in influencing plant defense [34]. A short term exposure to high temperatures (heat shock), e.g., submerging plants in 48–49 °C water for 20 seconds (s) one day before Bgh inoculation, resulted in susceptibility of barley (*H. vulgare* cv. Ingrid) near-isogenic lines containing different resistance genes (*mlo5*, *Mlg*, *Mla12*). In genetically susceptible barley (cv. Ingrid WT), the heat shock further increased susceptibility to Bgh [35]. In addition, we have shown that a short term heat shock (49 °C for 45 s) partially suppresses symptomless nonhost resistance of barley to wheat powdery mildew (*B. graminis* f. sp. *tritici*) [36]. In some cases, however, short term exposure to elevated temperatures may increase resistance to plant pathogens. In barley (cv. Golden Promise), a 50 °C heat shock for 60 s induces resistance against Bgh [37,38]. On the other hand, more prolonged high temperatures seem to decrease plant disease resistance in barley. It has been presented that an exposure to 36 °C for 30 minutes (min), 60 min and 120 min durations prior to pathogen inoculation causes enhanced susceptibility to Bgh in both genetically resistant (containing the *mlo* gene) and susceptible barley cultivars [39]. Interestingly, an exposure to high temperatures for several days combined with ambient or high carbon dioxide (CO_2_) concentrations (450 ppm CO_2_/26–30 °C or 850 ppm CO_2_/26–30 °C) inhibited wheat powdery mildew (*B. graminis* f. sp. *tritici*) growth independent of CO_2_ levels, and no typical powdery mildew symptoms were observed [24]. In this case, however, the absence of powdery mildew symptoms is presumably not due to the activation of plant defense responses but to the inhibition of pathogen growth at constant high temperatures. In summary, we find contradictory data about how heat stress may influence defense responses of barley to Bgh, probably because the plant cultivar, the powdery mildew race as well as the time between heat treatment and inoculation may differentially affect the barley/powdery mildew relationship at high temperatures.

We hypothesized that the duration of heat stress may significantly influence barley–Bgh interactions and pre-exposure of plants to prolonged heat may dramatically increase their susceptibility to powdery mildew. In order to simultaneously assess the effects of short and long term heat exposure on resistance/susceptibility of barley to Bgh, the objective of the present study was to determine the influence of heat stress (28 and 35 °C) of different durations (from 30 s to five days) on barley defense responses to subsequent infections by Bgh in susceptible and resistant barley lines.

## 2. Materials and Methods

### 2.1. Plant Materials and Pathogen Inoculation

The following barley (*H. vulgare*) cultivars and breeding lines were used in our experiments: cv. Ingrid *Mlo;* cv. GK-Stramm; cv. Antonella; cv. KWS-Meridian; cv. Hanzi; cv. MV Initium; MvHV05-17; MvHV07-17; MvHV14-18; MvHV118-17. Plants developed from approximately 30 seeds sown into two pots per treatment were grown in versatile environmental chambers (20 °C, 16 h light/8 h dark photoperiod). To assess the host responses of the barley cultivars and breeding lines to barley powdery mildew, *B. graminis* f. sp. *hordei* (Bgh) race A6, artificial inoculation was performed. The Bgh race A6 used in this study was kindly supplied by Karl-Heinz Kogel (Justus Liebig University, Giessen, Germany). Bgh was maintained on susceptible host plants (cv. Ingrid *Mlo*) in versatile environmental chambers (20 °C, 60% relative humidity, 16 h photoperiod with a light intensity of 100 μmol m^−2^ s^−1^). For inoculation, conidia from heavily infected barley were dusted equally onto primary leaves of 7 day-old barley seedlings of the cultivars/lines listed above [36]. Inoculated plants showed an inoculation density of ca. 50 conidia mm^−2^. Bgh symptoms were evaluated visually 7 days after inoculation (DAI).

### 2.2. Heat Stress

To detect how heat stress influences powdery mildew infection, we artificially stressed a selected resistant (MvHV07-17) and susceptible (MvHV118-17) barley line prior to Bgh inoculation in versatile environmental chambers at 20 °C (control), 28 and 35 °C (heat stress) with a 16 h light/8 h dark photoperiod. The reason for selecting 28 and 35 °C is that these temperatures may quite often occur under field conditions in Central Europe typically in periods when barley is exposed to Bgh (i.e., in May and June). The duration of heat stress ranged from 30 s to 5 days (30 s, 1 min, 1 h, 2 h, 6 h, 24 h, 48 h, 120 h). For the longer (prolonged) heat stresses (24, 48 and 120 h), the temperature was decreased to 25 °C during the 8 h dark period. Powdery mildew inoculation of heat stressed plants was performed as described above, immediately after heat treatments. 

### 2.3. Evaluation of Bgh Symptoms

The formation of Bgh symptoms in leaves of inoculated plants was evaluated visually 7 days after inoculation. Disease severity was estimated as the percentage of area covered by powdery mildew symptoms per leaf. Inoculated primary leaves for each plant were evaluated. Three independent biological experiments were conducted and 360 plants per experiment were assessed.

### 2.4. Quantitative Analyses of Powdery Mildew Biomass and Plant Defense Gene Expression

To quantify Bgh biomass, leaf tissue samples (5 primary leaves from 5 individual plants randomly selected and pooled per treatment) were taken from plants at 7 DAI in liquid nitrogen following symptomatic evaluation. Samples for defense gene expression (5 primary leaves from 5 individual plants randomly selected and pooled per treatment) were taken at early time points (1, 2, 6 and 24 h) after heat stress or after heat stress immediately followed by Bgh inoculation, respectively, and stored at −70 °C. To analyze Bgh biomass and plant defense gene expression, a reverse transcription followed by quantitative real-time polymerase chain reaction (RT-qPCR) method was used. Collected leaf tissue was grounded in liquid nitrogen and total RNA (including plant and Bgh RNAs) was isolated by the Plant Total RNA Extraction Miniprep System Kit according to the manufacturer’s instructions (Viogene-Biotek Inc., Taipei, Taiwan). After RNA isolation, DNAse I treatment with RQ1 RNase-Free DNase was performed (Promega Inc., Madison, WI, USA). RNA quantity and quality (260/280 and 260/230 ratios) were assessed by a MaestroNano Spectrophotometer (Maestrogen Inc., Hsinchu City, Taiwan) and RNA degradation was also checked by formaldehyde agarose gel electrophoresis of total RNA. One-thousand ng total RNA was used for reverse transcription (RT) in each sample. RT was done with a RevertAid™ H^−^ cDNA Synthesis Kit (Thermo Fisher Scientific Inc., Waltham, MA, USA) according to the manufacturer’s instructions. For negative control, a pool of randomly selected RNA samples were applied to which no reverse transcriptase was added. The qPCR for assaying relative expression of Bgh *Glyceraldehyde 3-phosphate dehydrogenase* (*BgGAPDH*) and barley defense genes *BAX inhibitor-1* (*HvBI-1*), *Pathogenesis related-1b* (*HvPR1-b*) and *Respiratory burst oxidase homologue F2* (*HvRBOHF2*) along with the barley reference gene *Ubiquitin* (*HvUbi*) was conducted with the 2 × SYBR FAST Readymix reagent (KAPA Biosystems Inc., Wilmington, MA, USA). The qPCR reactions were conducted as described by Höller et al. [40]. In brief, the PCR reaction mix contained 7.5 μL KAPA SYBR FAST qPCR Master Mix (2X), 0.75 μL of 5 μM forward and reverse primers each, 3.5 μL PCR-grade water and 2.5 μL of 20-fold diluted cDNA in 15 μL total reaction volume. DNA amplifications were performed in a Bio-Rad CFX-96 real-time thermocycler (Bio-Rad Inc., Hercules, CA, USA), running a standard program (95 °C for 2 min, 40 cycles at 95 °C for 10 s, 60 °C for 10 s and 72 °C for 10 s), followed by a melting curve analysis to determine amplicon specificity using a temperature range from 65 to 95 °C with increments of 0.5 °C. Gene expression was normalized to a barley *Ubiquitin* gene (*HvUbi*) as a reference. Previous research has shown that *HvUbi* is a reliable reference gene for assaying gene expression changes in barley exposed to either powdery mildew infection, heat or drought stress [19,41,42,43,44]. The suitability of *HvUbi* as a reference gene was tested by analysis of cycle threshold (CT) variation in response to heat treatments and Bgh infection. Significant changes were not observed in CT values (mean ± standard deviation, SD) for *HvUbi* during treatments. All reactions were performed using three independent biological experiments with three technical replicates per biological sample. In each run, water-only controls and non-reverse-transcribed RNA were used as negative controls. The primer efficiencies for the genes tested were between 101–106%. Changes in gene expression were calculated using the 2^−ΔΔCT^ method [45]. For primers used in qPCR, see Table 1.

### 2.5. Statistical Analyses

Statistical analyses were carried out using the Statistica 13 software (TIBCO Software, Palo Alto, CA, USA). Powdery mildew coverage on the leaf and relative gene expression values were log transformed to achieve homogeneity of variances (assessed by Bartlett’s test). Analysis of variance (ANOVA) and Tukey’s post-hoc test were employed and differences at *p* < 0.05 were considered as statistically significant.

## 3. Results

### 3.1. Testing Different Barley Lines to Powdery Mildew Resistance

**Figure 1:**
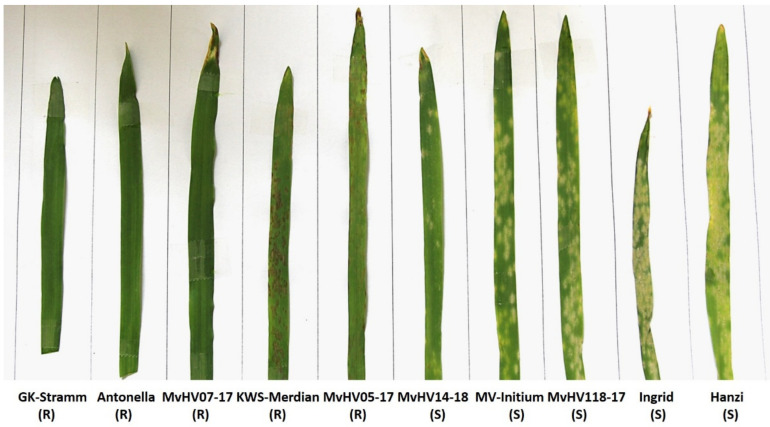
Absence or presence of powdery mildew (*Blumeria graminis* f. sp. *hordei* race A6; Bgh) symptoms in ten different barley cultivars and lines seven days after inoculation. Resistance or susceptibility of barley cultivars and lines to Bgh are marked with the letters (R) and (S).

In order to detect how barley (*H. vulgare*) plants respond to powdery mildew (*B. graminis* f. sp *hordei* race A6) infection at a physiologically optimal temperature (20 °C), ten different barley cultivars and breeding lines (cv. Ingrid WT, cv. GK-Stramm, cv. Antonella, cv. KWS-Meridian, cv. Hanzi, cv. MV Initium, MvHV05-17, MvHV07-17, MvHV14-18, MvHV118-17) were tested. Artificial inoculation of the barley plants mentioned above was performed with Bgh race A6 and the formation of powdery mildew symptoms in infected leaves was evaluated visually 7 days after inoculation (Figure 1). Disease severity was estimated as the percentage of area covered by powdery mildew symptoms per leaf. Our results showed that no visible powdery mildew symptoms were detectable on GK-Stramm, Antonella, MvHV07-17, KWS-Meridian and MvHV05-17. However, KWS-Meridian and MvHV05-17 displayed a hypersensitive response (HR) (i.e., resistance associated with localized necrotic lesions) during infection. In contrast, MvHV14-18, MV Initium, MvHV118-17, Ingrid WT and Hanzi cultivars showed different levels of susceptibility to Bgh with visible powdery mildew symptoms on leaves (Figure 1).

### 3.2. Determination of the Influence of Heat Stress on Powdery Mildew Infection

**Figure 2:**
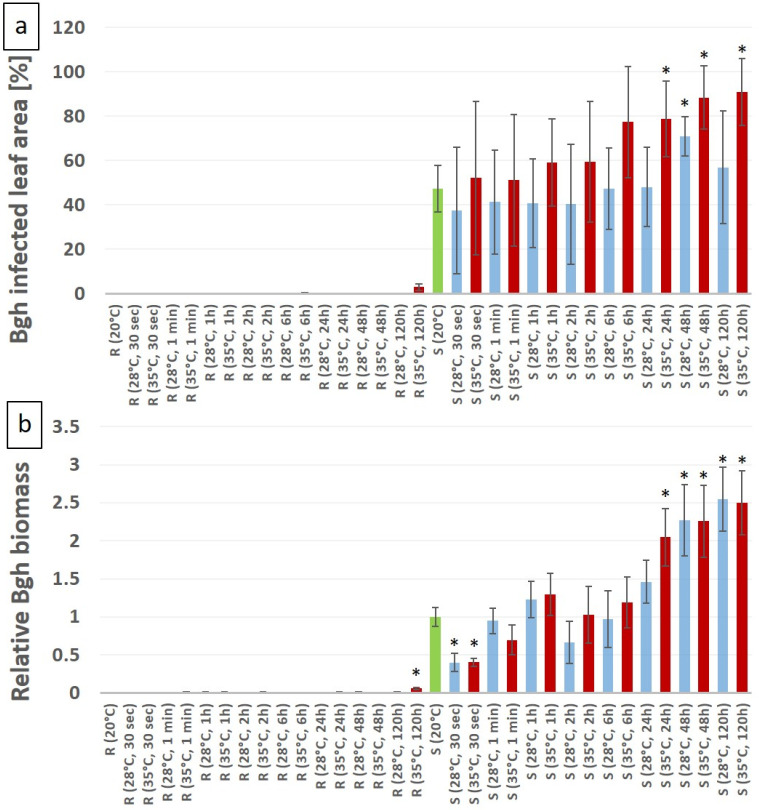
Evaluation of powdery mildew (*Blumeria graminis* f. sp. *hordei* A6, Bgh) symptom severity (**a**) in resistant (MvHV07-17; R) and susceptible (MvHV118-17; S) genotypes of barley seven days after inoculation. Barley plants were exposed to high temperature stresses (28 °C, blue columns and 35 °C, red columns) for different periods of time (30 s to 120 h) before powdery mildew inoculation. Symptom severity was calculated as the percentage of area covered by powdery mildew symptoms per leaf. Non-heat treated plants were held at 20 °C (green columns). Relative Bgh biomass (**b**) in resistant (MvHV07-17; R) and susceptible (MvHV118-17, S) genotypes of barley seven days after inoculation. Barley plants were exposed to high temperature stresses as described above for (**a**). The graphs show the average of three experiments. Error bars represent standard deviation. Asterisks (*) indicate statistically significant differences between non-heat treated and heat treated plants within the respective barley genotypes at *p* < 0.05.

To test how heat stress influences the defense responses of barley plants to Bgh, we selected two barley lines, one that displays no visible Bgh symptoms (MvHV07-17) and one susceptible line (MvHV118-17) which shows around 50% powdery mildew coverage per infected leaf at 20 °C. These barley lines were subjected to high-temperature pretreatment of various durations immediately before Bgh inoculation. Subsequently, disease symptoms were visually evaluated at seven DAI. Our results showed that the resistant barley line MvHV07-17 retained its resistance to the pathogen even after previous exposure to high temperatures based on the extent of powdery mildew symptoms at 7 DAI (Figure 2). In contrast, in the MvHV118-17 susceptible line there was a significant increase in the proportion of powdery mildew-covered area in plants previously exposed to 35 °C for 24, 48 and 120 h (Figure 2). However, no significant enhancement of powdery mildew symptoms was observed following heat exposure for less than 24 h at 35 °C. Furthermore, 28 °C heat stress enhanced susceptibility only at 48 h of heat treatment (Figure 2). In addition to the symptomatic assessment, the quantification of Bgh was also performed by RT-qPCR. These results were almost identical to the results of the symptom assessment; however, we found minor differences. As mentioned above, the resistant barley line MvHV07-17 retained its resistance to Bgh even after previous exposure to high temperatures; however, following a previous exposure to 35 °C for 120 h, the qPCR showed a significant increase in powdery mildew biomass, as compared to plants held at 20 °C (Figure 2). The Bgh biomass significantly increased in the susceptible MvHV118-17 line previously exposed to 35 °C for 24, 48 and 120 h; however, at 28 °C, a Bgh biomass increase was observed only following a 48 and 120 h of heat exposure. Interestingly, a short-term heat shock (30 s at 28 and 35 °C) significantly reduced the Bgh A6 biomass in MvHV118-17.

**Figure 3:**
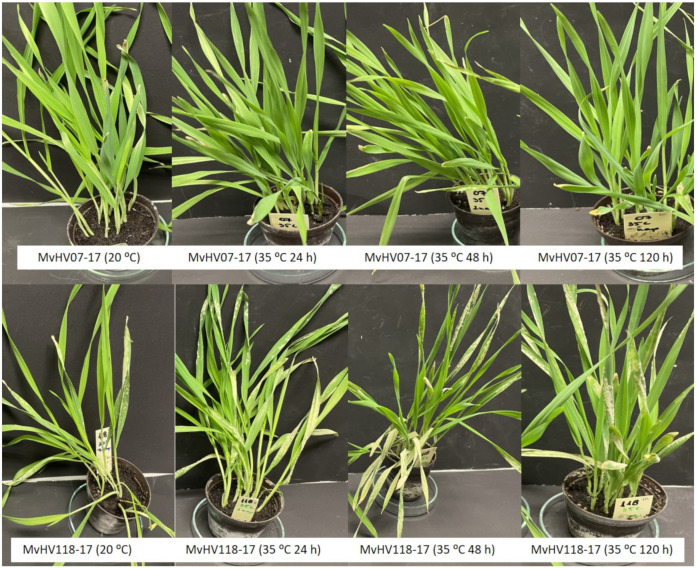
Powdery mildew (*Blumeria graminis* f. sp. *hordei* A6) symptoms in resistant (MvHV07-17) and susceptible (MvHV118-17) genotypes of barley seven days after inoculation. Plants were pretreated with heat (35 °C) for 24 h, 48 h and 120 h before powdery mildew inoculation. Control plants were kept at 20 °C.

Taken together, an enhancement of Bgh symptoms in the susceptible barley line (MvHV118-17) following exposure to 35 °C for 24, 48 and 120 h is detectable, as compared to the plants held at 20 °C. However, no Bgh symptoms were detectable in the resistant line (MvHV07-17), even at high temperatures (Figure 3). Based on the above results, long term (24, 48 and 120 h) heat stress at 35 °C significantly increased Bgh symptoms and biomass in the susceptible barley line MvHV118-17. In contrast, a short-term heat shock (30 s at 35 °C) did not influence Bgh symptoms but significantly reduced the Bgh biomass in line MvHV118-17 (Figure 2).

### 3.3. Expression of Plant Defense/Stress Genes in Heat-Stressed and BGH-Infected Barley

**Figure 4:**
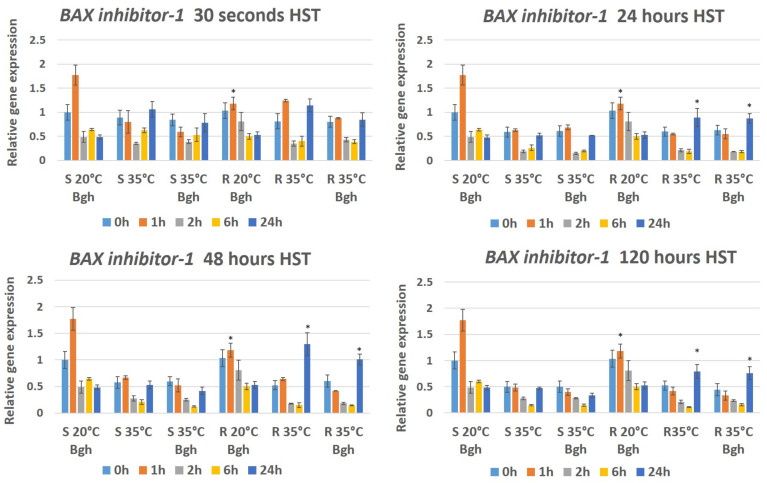
Expression of the barley *BAX inhibitor-1* gene as detected by RT-qPCR in resistant MvHV07-17 (R) and susceptible MvHV118-17 (S) barley lines at early time points (0 h, 1 h, 2 h, 6 h and 24 h) following powdery mildew (Bgh) inoculation. Heat shock treatments (HST) at 35 °C for 30 s, 24 h, 48 h and 120 h were applied immediately before powdery mildew inoculation (R 35 °C Bgh and S 35 °C Bgh). Heat-treated but not inoculated (R 35 °C and S 35 °C) and inoculated but not heat-treated plants (R 20 °C Bgh and S 20 °C Bgh) were used as controls. The graphs show the average of three experiments. Error bars represent standard deviation. Asterisks (*) indicate statistically significant differences between resistant and susceptible lines for each specific treatment at *p* < 0.05.

To assess the possible combined effects of heat stress and powdery mildew infection on the activities of stress/defense-associated genes (*BAX inhibitor-1*, *Pathogenesis related 1-b* and *Respiratory burst oxidase homologue F2*), we assayed their expression in both resistant and susceptible plants inoculated with powdery mildew and previously exposed to high temperatures. As controls, defense gene expression was also assayed in plants that received only heat treatment but no Bgh inoculation and inoculated plants that were held at an optimal temperature (20 °C). In the Bgh-inoculated susceptible line MvHV118-17 (S) held at 20 °C, the expression of *BAX inhibitor-1* (*HvBI-1*) increased significantly one hour after inoculation and then gene expression was dropped to ca. half within 24 h, as compared to the 0 h control. The expression of *HvBI-1* in resistant MvHV07-17 (R) plants showed a similar trend as in the susceptible plants except that at 1 h we did not experience a significant increase in gene expression (Figure 4).

**Figure 5:**
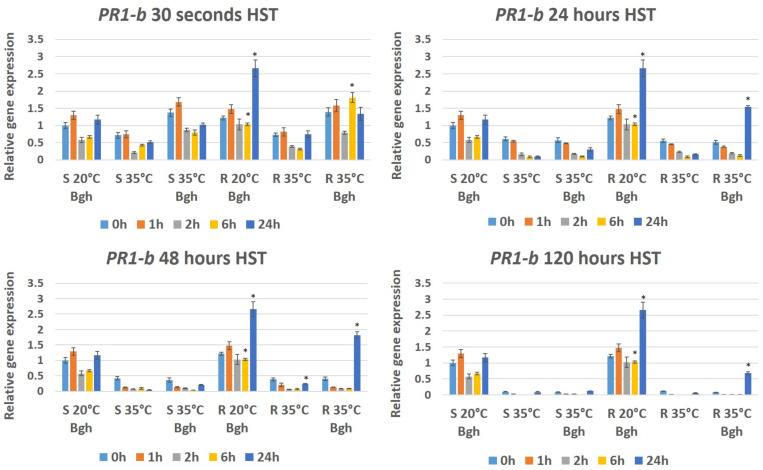
Expression of the barley *Pathogenesis related-1b* gene (*PR1-b*) as detected by RT-qPCR in resistant MvHV07-17 (R) and susceptible MvHV118-17 (S) barley lines at early time points (0 h, 1 h, 2 h, 6 h and 24 h) following powdery mildew (Bgh) inoculation. Heat shock treatments (HST) at 35 °C for 30 s, 24 h, 48 h and 120 h were applied immediately before powdery mildew inoculation (R 35 °C Bgh and S 35 °C Bgh). Heat-treated but not inoculated (R 35 °C and S 35 °C) and inoculated but not heat-treated plants (R 20 °C Bgh and S 20 °C Bgh) were used as controls. The graphs show the average of three experiments. Error bars represent standard deviation. Asterisks (*) indicate statistically significant differences between resistant and susceptible lines for each specific treatment at *p* < 0.05.

Long-term (24, 48 and 120 h) heat stress significantly reduced the expression of *HvBI-1* in both lines at zero, one, two and six h after Bgh inoculation. The decrease in gene expression was detectable in both infected and uninfected plants (Figure 4). Interestingly, 24 h after inoculation, *HvBI-1* expression increased significantly only in the resistant line (MvHV07-17), regardless of infection, to levels comparable to *HvBI-1* expression in control plants maintained at 20 °C (Figure 4). A short-term heat shock (30 s) generally did not cause a marked decrease in gene expression; however, 24 h after heat stress and/or inoculation the expression of *HvBI-1* in heat stressed or heat stressed and infected plants increased significantly in both resistant and susceptible lines (Figure 4). In the Bgh-inoculated, susceptible MvHV118-17 (S) line kept at 20 °C, the expression of *Pathogenesis related 1-b* (*HvPR1-b*) overall did not change significantly during the first 24 h of infection; however, in the resistant line (MvHV07-17), the expression of *HvPR1-b* doubled as compared to the zero-hour plants (Figure 5). Heat stress significantly reduced the expression of *HvPR1-b* in both lines at all examined time points after HST (Figure 5). As the duration of heat stress increased, a dramatic suppression of *HvPR1-b* expression became clearly evident. While the 30-s heat stress reduced *HvPR1-b* expression by only ca. half, the 120 h heat stress reduced expression to 1/100 of control levels (Figure 5). Remarkably, however, the reduced expression of *HvPR1-b* in heat-treated plants returned to normal or higher levels only in the infected resistant line (MvHV07-17), 24 h after heat stress and inoculation (Figure 5).

**Figure 6:**
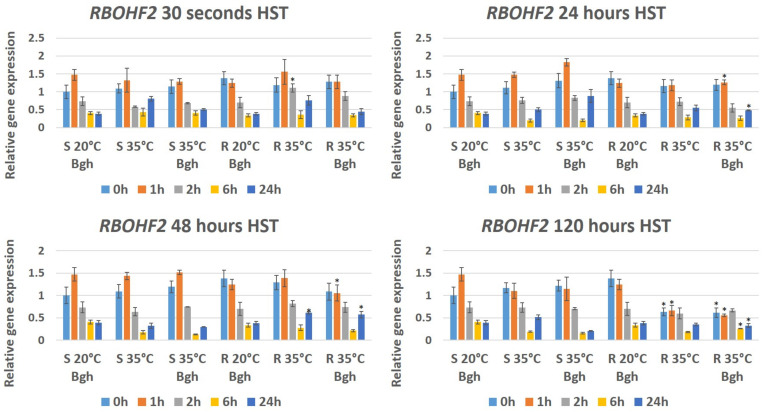
Expression of the barley *Respiratory burst oxidase homologue F2* gene (*RBOHF2*) as detected by RT-qPCR in resistant MvHV07-17 (R) and susceptible MvHV118-17 (S) barley lines at early time points (0 h, 1 h, 2 h, 6 h and 24 h) following powdery mildew (Bgh) inoculation. Heat shock treatments (HST) at 35 °C for 30 s, 24 h, 48 h and 120 h were applied immediately before powdery mildew inoculation (R 35 °C Bgh and S 35 °C Bgh). Heat-treated but not inoculated (R 35 °C and S 35 °C) and inoculated but not heat-treated plants (R 20 °C Bgh and S 20 °C Bgh) were used as controls. The graphs show the average of three experiments. Error bars represent standard deviation. Asterisks (*) indicate statistically significant differences between resistant and susceptible lines for each specific treatment at *p* < 0.05.

As mentioned before, short-term (30 s) HST reduced the expression of *HvPR1-b*; however, a 30 s HST and Bgh inoculation together increased the expression of the gene at most investigated time points in both barley lines (Figure 5). In both the susceptible (MvHV118-17) and resistant (MvHV07-17) Bgh-inoculated lines held at 20 °C, the expression of *HvRBOHF2* was reduced 2, 6 and 24 h after inoculation (Figure 6). Interestingly, however, a 30 s HST induced *HvRBOHF2* expression in both lines 24 h after HST but only in non-inoculated plants (Figure 6). Long-term (48 and 120 h) HST significantly reduced the expression of *HvRBOHF2* at 6 h after HST in both barley lines. However, after a 120 h HST, a reduced expression of *HvRBOHF2* was detectable both at 6 and at 24 h after treatments but primarily in the susceptible line upon Bgh inoculation (Figure 6). In resistant plants (MvHV07-17) heat-treated for 48 h, *HvRBOHF2* expression began to increase at 24 h after heat stress regardless of Bgh inoculation, a similar trend as seen with *HvBI-1* (Figure 6).

## 4. Discussion

Heat stress may significantly influence plant–pathogen interactions. To our knowledge, this is the first report showing that pre-exposure of powdery mildew-susceptible barley (MvHV118-17) to a prolonged (24, 48, 120 h) heat stress enhances susceptibility to the powdery mildew *Blumeria graminis* f. sp *hordei* (Bgh), manifested as both increased symptom severity and pathogen levels. Remarkably, in the resistant barley line (MvHV07-17), heat stress had no significant effect on either symptoms or pathogen levels; only the longest duration of high temperature stress (120 h at 35 °C) caused a marginal increase in pathogen levels, suggesting that resistance of MvHV07-17 to Bgh could be durable even under field conditions. However, previous studies demonstrate that prolonged heat stress often suppresses plant disease resistance to various pathogens, likely due to heat-induced conformation changes in the protein products of plant resistance/defense genes [23,34,46,47,48,49,50,51]. It remains to be elucidated whether the effective Bgh resistance of MvHV07-17 barley is due to efficient repair or evasion of conformational changes in resistance and defense-related proteins.

The role of short-term (from 30 s to 2 h) heat stress in modulating powdery mildew resistance in cereals had been studied previously. A short-term heat shock (30–40 s at 50 °C) followed by immediate Bgh inoculation significantly reduced powdery mildew infection in susceptible barley [37,38] similar to our results, where a 30 s heat shock at 28 and 35 °C also significantly reduced Bgh accumulation in susceptible MvHV118-17. On the contrary, others have shown that a short term exposure to high temperature, e.g., submerging plants in 49 °C water for 20 s, increased susceptibility of near-isogenic barley lines to Bgh. In genetically susceptible barley, the heat-shock further increased susceptibility, while in powdery mildew resistant barley lines resistance was converted into susceptibility [35]. In this case, however, Bgh inoculation was administered one day after heat-shock, as opposed to our work and previous studies, where heat shock was directly followed by inoculation [37,38]. It seems that the time elapsed between heat shock and Bgh inoculation is an important factor in determining the final outcome of infection (resistance vs. susceptibility) that could explain the different results of the above mentioned studies. In barley, Schwarzbach [39] has shown that exposure to a high temperature (36 °C for 30 min, 60 min and 120 min durations) causes enhanced susceptibility to Bgh in both genetically resistant (*mlo*) and susceptible barley cultivars [39]. In contrast, we found that exposure of barley to 35 °C for 60 or 120 min did not cause any significant effect on Bgh symptoms or biomass in both lines 7 days after inoculation. Importantly, in order to better simulate field conditions, we used intact barley plants in our assays, as opposed to the study of Schwarzbach [39], where leaf segments on agar medium were employed, a possible cause of differential reactions of heat-stressed barley (35–36 °C for 30–120 min) to powdery mildew infection.

Our results show that the expression of three plant defense-related genes (*BAX inhibitor-1*, *Pathogenesis related 1-b* and *Respiratory burst oxidase homologue F2*) is repressed drastically during prolonged (24, 48 and 120 h) heat stress. Interestingly, however, the expression of *BAX inhibitor-1* and *Respiratory burst oxidase homologue F2* is quickly restored (24 h after heat stress) in the resistant plants regardless of Bgh infection but not in the susceptible barley line. Furthermore, repression of the *Pathogenesis related 1-b* gene was also reactivated after heat stress but only in Bgh-inoculated resistant plants.

BAX Inhibitor-1 (BI-1) is a programmed cell death (PCD) suppressor in eukaryotes [52,53]. *Arabidopsis BI* mutants show increased sensitivity to heat shock-induced cell death; however, the mutants were indistinguishable from wild-type plants under normal growth conditions [54]. Furthermore, overexpressing the pepper (*Capsicum annuum* L.) gene *CaBI-1* in tobacco (*Nicotiana tabacum* L.) markedly improved tolerance to high temperature, water deficit, and high salinity in transgenic plants [55]. It can be assumed that heat stress induced PCD in Bgh-resistant barley (MvHV07-17) is inhibited by *HvBI-1* which is reactivated only in this line within 24 h after the heat shock. Although *HvBI-1* expression was enhanced after powdery mildew inoculation in different barley lines, *HvBI-1* expression was most pronounced in resistant genotypes (*Mla12*, *Mlg*) undergoing a hypersensitive resistance (HR) associated with PCD [56]. These authors hypothesized that BI-1 is suppressing cell death in plant tissues mounting an HR after fungal attack [56]. Furthermore, it was shown that barley BI-1 is a Bgh susceptibility factor, an effect likely caused by PCD suppression, since powdery mildews are biotrophic pathogens that prefer live host tissues for efficient infection [57,58,59]. Our results showed that the resistant MvHV07-17 barley kept at optimal temperatures (20 °C) did not show any visible HR symptoms after Bgh inoculation. Presumably, no HR (PCD) develops and therefore an increase in *HvBI-1* expression is not detectable in infected plants. However, BI-1 may have a role not only in PCD-inhibition but—at least in certain plant–pathogen interactions—in disease resistance *per se*, which has been shown to be the case in wheat, where silencing of *BI-1* resulted in converting an HR-type resistance to the rust pathogen *Puccinia striiformis* f. sp. *tritici* to partial susceptibility [60]. Furthermore, silencing of *BI-1* in *N. benthamiana* enhanced systemic accumulation of *Potato virus X* and *Potato virus Y* [61]. Nevertheless, our results suggest that enhanced *HvBI-1* expression in the Bgh-resistant barley line MvHV07-17 has a role primarily in alleviating heat stress and PCD, rather than influencing pathogen resistance.

Prolonged heat stress reduced expression of the barley *Respiratory burst oxidase homologue F2* gene (*HvRBOHF2*) primarily in the susceptible line, while *HvRBOHF2* expression was more or less restored (24 h after heat stress) in resistant plants, regardless of Bgh inoculation. *RBOH* genes encode plasma membrane proteins with NADPH-oxidase activity and RBOH-dependent reactive oxygen species (ROS) generation is associated with pathogen recognition during the oxidative burst [62]. Transgenic barley *HvRBOHF2*-knock down seedlings were much more susceptible to penetration caused by Bgh [43,63], pointing to the role of *HvRBOHF2* in barley Bgh resistance. Interestingly, suppression of resistance to *Tobacco mosaic virus* (TMV) at higher temperatures (30 °C) is also correlated with reduced ROS generation and down-regulation of expression of *NtRBOHD*, a tobacco functional homolog of *HvRBOHF2* [64]. This suggests that a reduced expression of *HvRBOHF2* in the barley line MvHV118-17 exposed to prolonged heat stress may underpin not only Bgh-susceptibility but also heat sensitivity. Indeed, transcript levels of ROS-producing *RBOH* genes decrease in response to heat (42 °C) in Arabidopsis [65]. Importantly, the activity of *RBOH*-encoded NADPH-oxidases have a dual role during abiotic and biotic stresses by producing ROS and initiating PCD in infected/stressed plant cells and simultaneously limiting the spread of cell death in adjacent cells by, e.g., activating antioxidant enzymes [7,8]. Accordingly, an increased NADPH-oxidase activity 24 h after powdery mildew infection is correlated with a symptomless (i.e., suppressed PCD) nonhost resistance of barley to *B. graminis* f. sp. *tritici*, suggesting that barley *RBOH* genes may confer both pathogen resistance and PCD limitation [36]. In fact, *HvRBOHF2* has been shown to negatively regulate PCD in Bgh-infected, older (17 day-old) barley leaves, while conferring Bgh resistance primarily in younger (11 day-old) leaves [63]. Therefore, a suppressed *HvRBOHF2* transcription in the Bgh-susceptible MvHV118-17 barley may indeed contribute to increased sensitivity to heat stress-induced PCD while also conferring Bgh susceptibility.

The activation of *PR1* genes and accumulation of their protein products in plants during pathogen attack is well-known [13]. Enhanced expression of different *PR* genes including *PR1* is induced in resistant wheat plants infected with powdery mildew but not in susceptible cultivars [66]. Moreover, plant PR1 proteins have sterol binding activity and hindering fungal sterol biosynthesis can be also potentially effective against powdery mildew fungi [13]. It is known that the barley PR1-b protein contributes to penetration resistance to Bgh, since transient silencing of *PR1-b* increases penetration efficiency of the pathogen in attacked epidermal cells [12]. Furthermore, another PR protein, PR17c is also required for penetration resistance of barley to powdery mildew [67]. A Bgh effector candidate, CSEP0055 interacts with barley PR1-b and PR17c leading to enhanced virulence, indicating a possible suppression of these PR proteins by Bgh [67]. In the present study, we have shown that a prolonged heat stress drastically reduces the expression of *HvPR1-b* in both resistant and susceptible barley lines. However, the resistant line (MvHV07-17) retained its ability to defend itself against Bgh even following a pre-exposure to high temperatures (35 °C), possibly, at least in part, by the rapid recovery of *HvPR1-b* gene expression in Bgh-infected plants, which confirms the role of barley PR1-b as a pivotal component of powdery mildew resistance.

## 5. Conclusions

We found that pre-exposure to a prolonged heat stress (35 °C for 24, 48 and 120 h) enhances powdery mildew infection in the susceptible barley line MvHV118-17. This suggests that with an increased likelihood of sustained high temperatures under field conditions, an increased damage caused by powdery mildew infection of, e.g., cereals like barley can be expected. In contrast, we found that a powdery mildew resistant barley line (MvHV07-17) retained its resistance even at high temperatures, since only an exposure to 35 °C for 120 h could induce a marginal increase in powdery mildew biomass. Importantly, even such a mild perturbation of pathogen resistance in a genetically resistant crop line following a prolonged exposure to high temperatures may potentially contribute to an epidemic by providing an additional inoculum source in the field. Therefore, an important task for plant breeders should be to investigate any possible changes in pathogen (e.g., powdery mildew) resistance of a given crop line/cultivar in response to prolonged heat stress. In order to address this problem, plant breeding for tolerance to combined (i.e., abiotic and biotic) stresses will be a pivotal task in the near future. In fact, the powdery mildew resistant barley line used in this study (MvHV07-17) has been shown to exhibit enhanced drought tolerance, as compared to the susceptible line MvHV118-17 [68]. To create crop cultivars that simultaneously evade abiotic stresses (e.g., high temperature, drought) and resist pathogen infections, two key approaches to consider are: (1) introgression of defense related genes from wild species via conventional breeding or genetic engineering [69], and (2) isolating mutants that retain disease resistance during exposure to abiotic stresses like heat [50]. Therefore, deciphering temperature-sensitive defense mechanisms and identifying novel, robust plant resistance pathways will be likely one of the most important weapons against plant pathogens to minimize crop yield losses in response to global warming [23].

## Figures and Tables

**Table 1:** Oligonucleotide primers used in qPCR.

Accession Number	Gene	Sequence 5′-3′		Amplicon Length	Primer Efficiency
CAUH01004767	*Glyceraldehyde 3-phosphate dehydrogenase* (*BgGAPDH*)	F	GGAGCCGAGTACATAGTAGAGT	105 bp	106%
		R	GGAGGGTGCCG-AAATGATAAC		
M60175	*Ubiquitin* (*HvUbi*)	F	ACCCTCGCCGA- CTACAACAT	240 bp	102%
		R	AGTAGTGGCGGTCGAAGTG		
AJ290421	*BAX inhibitor-1* (*HvBI-1*)	F	ATGTTCTCGGTGCC- AGTCT	409 bp	101%
		R	GGCGTGCTTGATGTAGTC		
X74940	*Pathogenesis related -1b* (*HvPR1-b*)	F	GGACTACGACTACGGCTCCA	150 bp	104%
		R	GGCTCGTAGTTGCAGGTGAT		
EU566856.1	*Respiratory burst oxidase homologue F2* (*HvRBOHF2*)	F	TGCTCGGTCAGCACT	175 bp	105%
		R	TCCGCAATA GAACACTCC		

Abbreviations: F, forward primer; R, reverse primer.

## Data Availability

Not applicable.
